# Clinical presentations of acute pulmonary embolism: A retrospective cohort study

**DOI:** 10.1097/MD.0000000000034224

**Published:** 2023-07-14

**Authors:** Moshe Khasin, Ivan Gur, Elite Vainer Evgrafov, Kohava Toledano, Ronen Zalts

**Affiliations:** a Rambam Medical Center, Haifa, Israel; b The Ruth and Bruce Rappaport Faculty of Medicine, Technion Israel Institute of Technology, Haifa, Israel.

**Keywords:** clinical presentation, presenting symptoms of PE, pulmonary embolism (PE)

## Abstract

We aimed to investigate whether the unusual clinical presentation of pulmonary embolism (PE) varies by the type of provocation. In this retrospective cohort study, we examined the electronic health records (EHR) records of all patients diagnosed with PE (upon presentation or during hospitalization) presented to our tertiary hospital during 2014 to 2019. Inclusion criteria were the diagnosis of acute PE and age above 18 years. Excluded were all patients to whom complete EHR were not available. The primary outcome was the main presenting symptom, categorized by a multidisciplinary consensus expert committee as either typical or atypical of PE. Comorbidities, vital signs, medications and laboratory results on presentations were recorded. 591 patients were included in the final analysis. Dyspnea was significantly less common and hemoptysis and chest pain more common in the unprovoked PE group (35%, 5%, and 25%, respectively) compared with nonmalignant (42.6%, 0%, and 16.3%) and malignancy-associated (47.7%, 0.9%, and 8.2%) PE (Pv = 0.02, 0.002 and 0.001, respectively). No recorded symptoms were the third most common presentation overall, accounting for a significantly (Pv < 0.001) higher proportion of PE patients with malignancy (19%) whereas atypical presentation was the hallmark of patients with nonmalignant provokation (19.7%) (Pv = 0.005). Accounting for multiple potential confounders, the risk of atypical or asymptomatic presentation was higher with lower heart rates (RR = 0.974 95%C.I. [0.957–0.990]) and higher pulse oximetry saturation (RR = 1.114 95%CI [1.034–1.201]). The clinical presentation of PE varies with different types of provoking factors, with atypical presentation most common in nonmalignant provocation and asymptomatic presentation most prevalent in patients with underlying malignancy. Further studies are needed to determine the effect of said variance on long term clinical outcomes.

## 1. Introduction

Pulmonary Embolism (PE) is a form of potentially fatal venous thromboembolism (VTE). Accounting for at least 500,000 deaths annually in the developed world alone. PE that is not promptly diagnosed and treated is associated with over 30% short term mortality. The majority of deaths from PE occur within the first week. Treatment with anticoagulation reduces the direct mortality attributed to PE by 5 to 10-fold. Thus, the prompt diagnosis of PE is of utmost importance to patient survival.^[1]^

Often appellate “the great masquerader,” PE is famously vacillating in presentation.^[2]^ Symptoms of dyspnea, pleuritic chest pain, cough, and hemoptysis are often cited as typical presenting symptoms, while tachypnea and tachycardia have been found to be the most common presenting signs.^[3]^ Many other symptoms and signs, including syncope and various mental state changes, have been stipulated in the clinical presentation of PE.^[4]^ As an example, The implementation of fibrin degradation products, such as D-dimer, over the 1990s, saw an almost 2-fold increase in the incidence of PE, suggesting the low specificity of clinical presentation alone in the diagnosis of PE.^[5]^

However, previous systematic reports of acute PE found the presentation with none of the abovementioned typical signs and symptoms to be exceedingly rare, accounting for <2% to 5% of cases. In these studies, the incidence of asymptomatic presentation is either not specified or very low.^[1,3,4,6–8]^

Many conditions have been shown to significantly increase the risk of PE. These include active malignancy, prolonged immobilization (particularly trauma or surgical patients recovering from major abdominal or orthopedic surgery), and other inherited (e.g., various thrombophilia) or acquired (e.g., nephrotic syndrome) conditions.^[9]^ While the overall incidence of PE amongst patients with known active malignancy is estimated in the 10% range, certain malignancy types bear much higher lifetime risk of thromboembolism, with some series reporting up to 50% prevalence on autopsy.^[10]^ Whereas the incidence and prevalence of PE is well described in patients with malignancy as well as other types of provocation, paucity of evidence exists as to the clinical presentation attributes in these special populations.^[8,9,11]^ In particular, the rare and unusual clinical presentations in patients with malignancy or other predisposing factors for PE have not been systematically studied or reported.

In this study we aimed to systematically describe the presentation of acute PE clustered by known provocation status, with particular attention to the less common and atypical presentations.

## 2. Methods

This retrospective cohort study was conducted in Rambam Health care Campus (RMC), a tertiary level care 1100 beds medical center, situated in Haifa, northern Israel. The Electronic Health Registry (EHR) files of all patients hospitalized with the diagnosis of PE between January 1st 2014 and December 31st 2019 were reviewed.

All adult patients (18 years of age or older), newly diagnosed within any of the departments (including outpatient visits and ambulatory imaging done for any indication) in RMC during the study period were included. Exclusion criteria were: lack of fully available previous diagnoses list in the EHR available to us patients referred to RMC more than 48 hours after the initial diagnosis of PE.

Physician notes, admission and discharge reports, imaging interpretations and background diagnoses were manually and individually reviewed for each patient included in this study. Additional demographic, clinical and laboratory data, including date of birth, vital signs and laboratory results upon presentation were mined using the MD-Clone interface (version 3.01 or older). Machine mined data was assessed for accuracy and relevance by the investigator reviewing the EHR.

All presenting symptoms were categorized independently, based on the review of available literature, by 2 experienced internists (EW and KT) as typical or atypical. Any discrepancies were mitigated by a 5 member board certified experts’ consensus meeting including a pulmonologist, a critical care specialist, a rheumatologist, and 2 general internists. This multidisciplinary team also consolidated all cases into 1 of 3 provocation groups: malignancy related PE, non-malignancy provoked PE, and unprovoked PE. Any patient with known active malignancy of any sort within 6 months from the diagnosis of PE were categorized as malignancy related PE. Other etiologies were the presence of any of the following: within 1 month after a fracture or an orthopedic surgery (up to 6 weeks for femoral head fractures); 3 or more days of immobilization; long haul (over 8 hours airborn time) flight; Known thrombophilia; Peripartum. This study was reviewed and approved by the RMC institutional ethics committee (RMB-20-0223).

### 2.1. Statistical analysis

Standard descriptive statistics were used to summarize population characteristics. We used a chi-square test for categorical variables, Mann-Whitney *U* test for nonparametric variables and student unpaired *t* test for normally distributed continuous variables. Tukey correction was applied when applicable to adjust for multiple comparisons. Categorical variables were described using proportions and percentages, non-parametric variables with median and interquartile range and normally distributed continuous variables as mean with standard deviation.

Multivariate logistic regression modeling was performed using Pearl and Reed method. We used the Pearson correlation coefficient to determine possible correlations between independent variables, only variables not co-related (Pv > 0.1 on univariate analysis) were included in the model. A 2-sided Pv < 0.05 was considered statistically significant for all tests. All calculations were performed using SPSS software version 24.0.

## 3. Results

**Figure 1.**
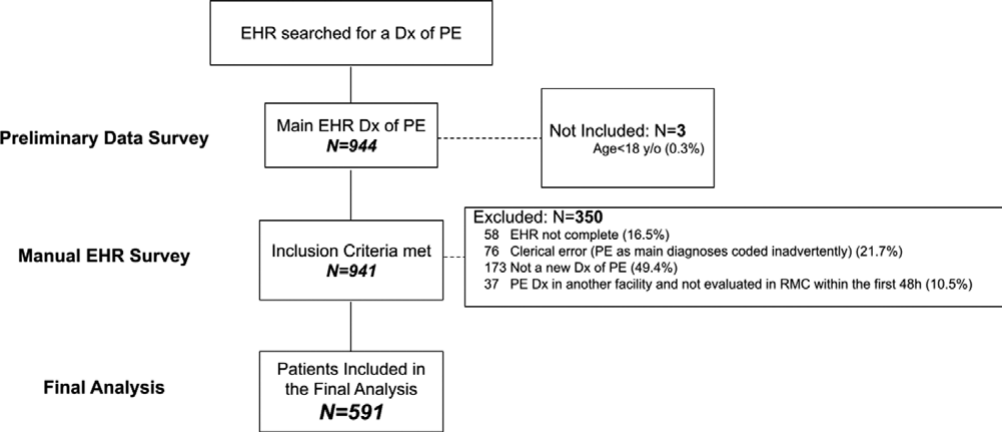
Study design. The study phases are presented in accordance with the CONSORT guidelines. Dx = diagnosis, EHR = electronic healthcare registry, PE = pulmonary embolism.

Of 944 electronic health registries with a main diagnosis of new PE during the study period, 3 were under the age of 18 and 350 met one or more exclusion criteria. Most (83.4%) of these were excluded due to the time elapsed from the initial PE diagnosis to the first evaluation within RMC, which would have befuddled the accuracy with which the initial clinical presentation was reflected in the EHR available to this study. Consequently, a cohort of 591 patients were included in the final analysis. A Consolidated Standards of Reporting Trials (CONSORT) diagram summarizing the data mining and filtering process is presented in Figure 1.

Patient age was significantly (Pv = 0.001) higher in patients with known malignancy or unprovoked PE (65 ± 12 years old) when compared to patients with other provoking factors (58.7 ± 21). No significant gender disparities were noted. The rate of hypertension was highest in the unprovoked group (51.7%), than those with malignancy (44.5%), and lowest in those with other known etiologies (35.7%; Pv = 0.012). Similar trends were found in the prevalence of prior VTE diagnosis (15%, 10%, and 7%, respectively; Pv = 0.045), diabetes (25%, 19%, and 13%; Pv = 0.015) and dyslipidemia (42%, 33%, and 23%; Pv = 0.01). No significant differences were noted between these groups in the prevalence of other cardiovascular diseases (ischemic heart disease, heart failure, atrial fibrillation, stroke), pulmonary diseases, thyroid dysfunction. Whereas low-molecular weight heparin was used in higher rates in those with provoked PE (11% vs 2.9%, Pv < 0.0001), no significant difference was noted in the use of other antiplatelets, anticoagulants or cardiovascularly active agents between the groups. The baseline characteristics of patients presented with a new diagnosis of PE, grouped by provocation status (malignancy, other and unknown), are summarized in Table 1.

**Table 1:** Study patients characteristics.

Total = 591	Unprovoked (N = 242)	Malignancy related (N = 220)	Other etiologies (N = 129)	*P* value
**Age [yr]**	**65.1 (±18**)	**65.2 (±12**)	**58.7 (±21**)	**.001**
Female (%)	122 (50.4%)	125 (56.8%)	77 (59.7%)	.175
Cardiovascular disease (%)				
IHD	34 (14%)	26 (11.8%)	8 (6.2%)	.077
**HTN**	**125 (51.7%**)	**98 (44.5%**)	**46 (35.7%**)	**.012**
HF	8 (3.3%)	5 (2.3%)	7 (5.4%)	.289
AF	9 (3.7%)	10 (4.5%)	8 (6.2%)	.552
CVA	10 (4.1%)	4 (1.8%)	6 (4.7%)	.26
**VTE**	**37 (15.3%**)	**23 (10.5%**)	**9 (7%**)	**.046**
**CKD**	**22 (9.1%**)	**2 (0.9%**)	**7 (5.4%**)	**<.001**
Pulmonary disease				
COPD	18 (7.5%)	13 (5.9%)	4 (3.1%)	
ASTHMA	4 (1.7%)	2 (0.9%)	1 (0.8%)	
ILD	1 (0.4%)	1 (0.5%)	0 (0%)	
Metabolic/Endocrine disease				
**Diabetes Mellitus**	**62 (25.6%**)	**42 (19.1%**)	**17 (13.2%**)	**.015**
**Dyslipidemia**	**103 (42.6%**)	**73 (33.2%**)	**30 (23.3%**)	**<.01**
Hypothyroidism	18 (7.4%)	11 (5%)	9 (7%)	.543
Medications influencing Hemodynamics				
Beta Blockers	68 (28.1%)	66 (30%)	32 (24.8%)	.581
ACEI	46 (19%)	45 (20.5%)	20 (15.5%)	.517
ARB	28 (11.6%)	25 (11.4%)	10 (7.8%)	.479
Diuretics	38 (15.7%)	23 (10.5%)	15 (11.6%)	.217
MRA	6 (2.5%)	3 (1.4%)	3 (2.3%)	.673
Alpha-2 agonists	0 (0%)	3 (1.4%)	0 (0%)	.427
Alpha-1 Blockers	19 (7.9%)	18 (8.2%)	6 (4.7%)	.427
Calcium channel blockers	38 (15.7%)	39 (17.7%)	23 (17.8%)	.805
Antiplatelets				
Aspirin	56 (23.1%)	51 (23.2%)	20 (15.5%)	.173
Clopidogrel	18 (7.4%)	7 (3.2%)	7 (5.4%)	.130
DAPT	0 (0%)	5 (2.3%)	2 (1.6%)	.099
Anti coagulants				
**LMWH**	**7 (2.9%**)	**23 (10.5%**)	**16 (12.4%**)	<.001
DOACs	2 (0.8%)	2 (0.9%)	3 (2.3%)	.398
VKA	2 (0.8%)	2 (0.9%)	2 (1.6%)	.787

Baseline patient characteristics are presented by a provocation group. One way analysis of variance (ANOVA) was used for age and chi square for all other variables.

ACEi = angiotensin converting enzyme inhibitors, AF = atrial fibrillation, ARBs = angiotensin receptor blockers, CKD = chronic kidney disease, COPD = chronic obstructive pulmonary disease, CVA = cerebrovascular accident, DAPT = dual antiplatelet, DOACs = direct oral anticoagulants, HF = heart failure, HTN = hypertension, IHD = ischemic heart disease, ILD = interstitial lung disease, VKA = vitamin K antagonists, VTE = venous thromboembolism.

Reviewing the plethora of presenting symptoms, the following were deemed atypical by our multidisciplinary team of experts: palpitations, abdominal pain, traumatic injury, nausea or vomiting, non-accidental fall, seizures, flank pain, delirium, dysarthria, general deterioration, headache, back pain and weakness. This full range of symptoms is presented in Supplementary Table 2, http://links.lww.com/MD/J240.

**Figure 2.**
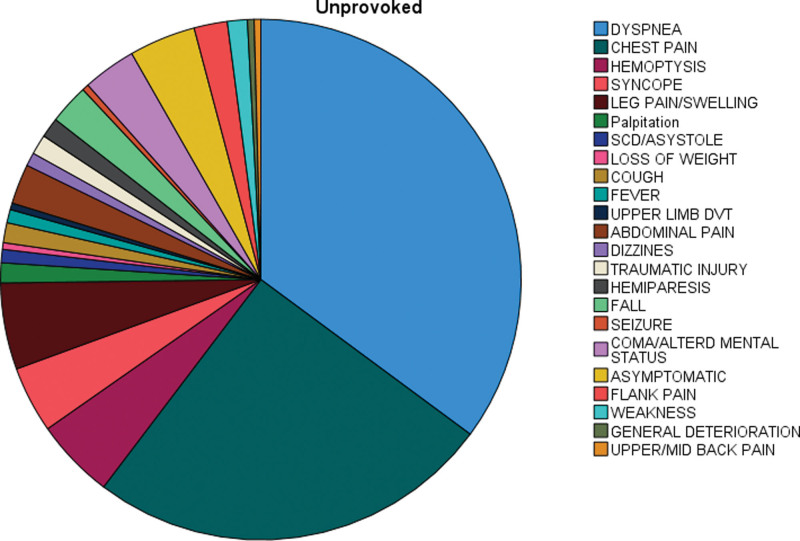
Presenting symptom (chief complaint) by final diagnosis. Displayed are the presenting symptoms of patients diagnosed with PE, by provokation group. DVT = deep vein throbosis, SCD = sudden cardiac death.

Dyspnea, the most common presenting symptom across all provokation groups, was significantly (Pv = 0.02) higher in patients with provoked PE (45.6%) compared to patients with unprovoked PE (35%). Conversely, hemoptysis was significantly rarer in the provoked vs. unprovoked PE groups (0.3% and 5%, respectively; Pv = 0.002). Chest pain was most prevalent in the unprovoked group, then nonmalignant provocation and lastly patients with malignancy (25%, 16%, and 8%; Pv < 0.001). Asymptomatic presentation was the third most common presentation overall, accounting for a significantly (Pv < 0.001) higher proportion of PE patients with malignancy (19%) compared to patients with unprovoked PE (4%) or other known etiologies (9%). On the other hand, atypical presentation was the hallmark of patients with nonmalignant provokation (19.7%) in which group such clinical presentation was significantly (Pv = 0.005) more common than in the known malignancy (7.3%) or no known provocation (11.2%). The rates of asymptomatic and atypical presentations, as well as the 5 most common presenting symptoms in this cohort are presented in Table 2 and in Figure 2.

**Table 2:** Clinical characteristics upon presentation.

Symptom	Total			Unprovoked			Malignancy related			Other etiologies			*P* value
	Order	N = 591	%	Order	N = 242	%	Order	N = 220	%	Order	N = 129	%	
Dyspnea	1	244	41.3	1	85	35.1	**1**	**105**	**47.7**	**1**	**55**	**42.6**	**.02**
Chest pain	2	100	16.9	**2**	**61**	**25.2**	**3**	**18**	**8.2**	**3**	**21**	**16.3**	**.001**
A- symptomatic	3	64	10.8	7	10	4.1	**2**	**42**	**19.1**	4	12	9.3	**.001**
Atypical symptoms	4	62	11.8	3	26	11.2	4	13	7.3	**2**	**23**	**19.7**	**.005**
Leg pain or swelling	5	29	4.9	4	13	5.4	5	10	4.5	5	6	4.7	.909
Syncope	6	17	2.9	6	10	4.1	14	2	0.9	6	5	3.9	.087
Hemoptysis	7	14	2.4	**5**	**12**	**5.0**	13	2	0.9		0	0	**.002**

The prevalence of each clinical presentation, along with relative order of frequency, is presented by the provocation group. Proportions were compared using Pearsons Chi Square, no cells had an expected count of <5.

Atypical Symptoms included: Palpitations, abdominal pain, traumatic injury, nausea or vomiting, non-accidental fall, seizures, flank pain, delirium, dysarthria, general deterioration, headache, back pain and weakness.

To assess for the possible predictors of a-typical or asymptomatic presentation, a multivariate logistic regression model was constructed. Heart rate at diagnosis was found to significantly decrease the propensity of non-classical symptomatology upon PE presentation (Relative Risk = 0.974 for each beat per minute, 95%C.I. [0.957–0.990]). Conversely, pulse oximetry O2 saturation upon presentation was found to significantly increase the likelihood of non-classical symptoms (RR = 1.114 with each 1% saturation, 95%C.I. [1.034–1.201]). No other exposure variables were found to independently predict non-classical presentation of PE with the accepted level of statistical significance (alpha = 0.05). These included vital signs on presentation (blood pressure and temperature), patient characteristics (gender, previous diagnoses of ischemic heart disease or heart failure, hypertension, stroke, kidney failure, liver or lung disease or diabetes) or chronic medications (beta, angiotensin receptor or calcium channel blockers, angiotensin converting enzyme inhibitors, mineralocorticoid receptor antagonists, diuretics, alpha blockers or agonists, antiplatelets and anticoagulants). This regression model is summarized in Supplementary Table 1, http://links.lww.com/MD/J239.

## 4. Discussion

In this study, patients with unprovoked PE were almost twice as likely to have had a prior diagnosis of VTE compared to patients with a known provocation factor. While the absolute incidence of previous VTE is higher than reported in some community-based series, other hospital based studies have described similar findings, suggesting some variation in patient selection as a result of the tertiary referral status of our medical center.^[3,12]^ The occurrence of VTE is undoubtedly a major predictor of recurrence, with the lifetime recurrence noted in 19% to 30% of patients with no identifiable causes of VTE. However, active investigation into the pathophysiology and specific etiology of recurrent VTE have resulted in highly heterogeneous, often conflicting findings.^[1,9]^ Furthermore, strong observational data suggests some forms of VTE (particularly those with strong identifiable risk factors including pregnancy or surgery associated with VTE) to bear minimal to no risk of recurrence.^[1]^ The limitations of the EHR available to us limited the reliable identification of the circumstances in which previous VTE was diagnosed. Nonetheless, this finding, in the context of the existent body of literature, supports the consideration of previous unprovoked VTE as a major risk factor - akin to other forms of provocations such as known thrombophilia, malignancy or immobilization.

Similarly, to previous studies patients with nonmalignant provocation were significantly younger.^[3,7]^ This is likely due to the very nature of provocation defining criteria, many of which (pregnancy, surgery) are most relevant in young, generally healthy individuals. This selection skewness is also likely to explain the lower prevalence of common chronic diseases, such as hypertension, diabetes or dyslipidemia, in the nonmalignant provocation group.

Asymptomatic presentation was significantly more common in patients with known underlying malignancy. This could be explained by 2 mutually complementary mechanisms. Firstly, the higher frequency of pulmonary imaging, archetypical of patients treated for active malignancy, creates an inherent propensity towards asymptomatic PE selection bias in this population.^[11,13]^ Secondly, previous investigators have suggested alternation in the physiologic response to pulmonary VTE in patients with malignancy. These include the lowering of hypoxic drive and the perception of dyspnea as well as alterations in pain perception.^[10]^

On the other hand, atypical presentation was most prevalent in patients with nonmalignant provocation. To the best of our knowledge the prevalence of atypical presentation was not previously reported. Moreover, we found no studies stratifying such incidence by provocation group. However, the body of existing knowledge could help illuminate our understanding of this important finding. Namely, some investigators have reported provoked PEs to be significantly smaller.^[8,11,12]^ While undoubtedly attributable at least in part to detection bias, a higher activation of counter regulatory pathways in the presence of chronic VTE could explain the lower burden of VTE in this population. This in turn would explain the lack of compensatory physiology and ensuing “typical” symptoms of chest pain and dyspnea.

Non classical (i.e., atypical or asymptomatic) presentation was significantly associated with lower heart rates and higher pulse-oximetry capillary oxygen saturation upon presentation. This finding was both statistically and clinically significant, withstanding correction for multiple potential confounders, including age and comorbidities, medical treatment affecting both VTE risk (anticoagulants and antiplatelets) and vital signs (e.g., adrenergic blockers), and laboratory results on presentation. This suggests a deviation from the common pathophysiology of “classical” PE in these patients. Perhaps more importantly, this finding could indicate a decreased sensitivity of the accepted diagnostic risk stratification tools (e.g., wells criteria, PE severity index, etc), all of which rely heavily on signs of heart rate and respiratory alterations.^[9]^

## 5. Limitations

This study has several important limitations. Firstly, the retrospective design inherently raises the risk of biases, particularly since no randomization was performed. Secondly, the lack of universally accepted criteria for PE suspicion and diagnostic work-up obscure the true incidence of PE. This is especially true for asymptomatic patients, and to a large extent in patients presenting with atypical symptoms, in many of whom PE was discovered incidentally. Lastly, data regarding the short and long term sequelae of PE (e.g., mortality, length of stay, etc) were not available for this analysis. Thus, we were unable to infer the prognostic significance of clinical presentation on short and long term clinical outcomes.

## 6. Conclusions

The clinical presentation of PE varies with different types of provoking factors. In this study, atypical presentation was more common in nonmalignant provocation, while asymptomatic presentation was most prevalent in patients with underlying malignancy. Coupled with evidence of altered signs in atypical and asymptomatic cases of PE (i.e., lower heart rates and higher O2 saturation), this raises questions as to the validity of our current approach to PE diagnosis in these populations. Further larger and preferably prospective studies are needed to determine the effect of clinical presentation, in particular - asymptomatic and atypical presentation of PE, stratified to the type of provocation, on clinically meaningful outcomes such as length of hospitalization, long term respiratory disability, and mortality.

## Author contributions

**Conceptualization:** Ivan Gur, Moshe Khasin, Ronen Zalts.

**Data curation:** Ivan Gur, Moshe Khasin, Ronen Zalts.

**Formal analysis:** Ivan Gur, Moshe Khasin, Ronen Zalts.

**Funding acquisition:** Ronen Zalts.

**Investigation:** Ivan Gur, Moshe Khasin, Elite Vainer Evgrafov.

**Methodology:** Ivan Gur, Moshe Khasin.

**Project administration:** Ivan Gur, Moshe Khasin, Elite Vainer Evgrafov.

**Resources:** Ivan Gur.

**Software:** Ivan Gur.

**Supervision:** Ivan Gur, Elite Vainer Evgrafov, Kohava Toledano, Ronen Zalts.

**Validation:** Ivan Gur, Kohava Toledano, Ronen Zalts.

**Visualization:** Ivan Gur, Moshe Khasin, Kohava Toledano, Ronen Zalts.

**Writing – original draft:** Ivan Gur, Ronen Zalts.

**Writing – review & editing:** Ivan Gur, Moshe Khasin, Elite Vainer Evgrafov, Kohava Toledano, Ronen Zalts.

## Supplementary Material




