# Supplementary material for: Clinical presentations of acute pulmonary embolism: A retrospective cohort study
Source: Medicine (Baltimore). 2023 Jul 14;102(28):e34224. doi: 10.1097/MD.0000000000034224 (PMC10344497; doi:10.1097/MD.0000000000034224)
Supplement: Supplementary file 1 [file medi-102-e34224-s001.pdf]

Supplementary Table 2 - Main Presenting Symptom

The chief complaint leading to PE diagnosis is presented by provokation group. *Abbreviations:*

Atypical Symptoms included: Palpitations, abdominal pain, traumatic injury, nausea or vomiting, non accidental fall, seizures, flank pain, delirium, dysarthria, general deterioration, headache, back pain and weakness

Total			Unprovoked			Malignancy			Other Provokation		
Symptom	N = 591	%	Symptom	N = 242	%	Symptom	N = 220	%	Symptom	N = 129	%
DYSPNEA	244	41.3	DYSPNEA	85	35.1	DYSPNEA	105	47.7	DYSPNEA	55	42.6
CHEST PAIN	100	16.9	CHEST PAIN	61	25.2	asymp	42	19.1	CHEST PAIN	21	16.3
**asymp**	64	10.8	LEG PAIN/ SWELLING	13	5.4	CHEST PAIN	18	8.2	asymp	12	9.3
LEG PAIN/ SWELLING	29	4.9	HEMOPTYSIS	12	5.0	LEG PAIN/SWELLING	10	4.5	LEG PAIN/SWELLING	6	4.7
SYNCOPE	17	2.9	SYNCOPE	10	4.1	cough	7	3.2	FLANK PAIN	6	4.7
HEMOPTYSIS	14	2.4	asymp	10	4.1	fever	6	2.7	SYNCOPE	5	3.9
**ABDOMINAL PAIN**	14	2.4	COMA/ALTERD MENTAL STATUS	8	3.3	ABDOMINAL PAIN	4	1.8	TRAUMATIC INJURY	5	3.9
**COMA/ ALTERD MENTAL STATUS**	14	2.4	ABDOMINAL PAIN	6	2.5	Dizziness	3	1.4	ABDOMINAL PAIN	4	3.1
**FLANK PAIN**	14	2.4	FALL	6	2.5	COMA/ALTERD	3	1.4	FALL	4	3.1

						MENTAL STATUS					
cough	11	1.9	FLANK PAIN	5	2.1	FLANK PAIN	3	1.4	COMA/ALTERD MENTAL STATUS	3	2.3
**FALL**	11	1.9	Palpitation	3	1.2	WEAKNESS generalized	3	1.4	fever	2	1.6
**fever**	10	1.7	cough	3	1.2	HEMOPTYSIS	2	0.9	upper/MID back pain	2	1.6
**TRAUMATIC INJURY**	9	1.5	TRAUMATIC INJURY	3	1.2	SYNCOPE	2	0.9	Palpitation	1	0.8
**WEAKNESS generalized**	7	1.2	HEMIPARESIS	3	1.2	Palpitation	2	0.9	cough	1	0.8
Palpitation	6	1.0	WEAKNESS generalized	3	1.2	upper limb DVT	2	0.9	NAUSEA / VOMITING	1	0.8
**Dizziness**	5	0.8	SCD/ASYSTOLE	2	0.8	loss of weight	1	0.5	WEAKNESS generalized	1	0.8
upper limb DVT	3	0.5	fever	2	0.8	TRAUMATIC INJURY	1	0.5	DYSPNEA	55	42.6
HEMIPARESIS	3	0.5	Dizziness	2	0.8	fatigue	1	0.5			
**upper/MID back pain**	3	0.5	loss of weight	1	0.4	NAUSEA / VOMITING	1	0.5			
SCD/ASYSTOLE	2	0.3	upper limb DVT	1	0.4	FALL	1	0.5			

loss of weight	2	0.3	SEIZURE	1	0.4	SEIZURE	1	0.5
**NAUSEA / VOMITING**	2	0.3	GENERAL DETERIORATION	1	0.4	drowsiness	1	0.5
**SEIZUREs**	2	0.3	upper/MID back pain	1	0.4	SHOULDER PAIN	1	0.5
fatigue	1	0.2						
drowsiness	1	0.2						
**GENERAL DETERIORATION**	1	0.2						
SHOULDER PAIN	1	0.2						
