# Supplementary material for: Clinical presentations of acute pulmonary embolism: A retrospective cohort study
Source: Medicine (Baltimore). 2023 Jul 14;102(28):e34224. doi: 10.1097/MD.0000000000034224 (PMC10344497; doi:10.1097/MD.0000000000034224)
Supplement: Supplementary file 2 [file medi-102-e34224-s002.pdf]

Supplementary Table 1 - Multivariate logistic regression

The summary of a multivariate regression model is presented hereby. The dependent variable is any non-classical presentation, including those with atypical or asymptomatic symptomatology upon PE diagnosis.

Logistic Regression Model									
Atypical or Asymptomatic presentation[a]		B	Std. Error	Wald	df	Sig.	Exp(B)	95% Confidence Interval for Exp(B)	
								Lower Bound	Upper Bound
1.00	Intercept	-27.081	7539.172	.000	1	.997			
	Age at diagnosis	.007	.011	.449	1	.503	1.007	.986	1.029
	HR	-.027	.009	9.384	1	.002	.974	.957	.990
	SYS_BP	.000	.008	.000	1	.995	1.000	.984	1.016
	DYAS_BP	.005	.014	.141	1	.707	1.005	.978	1.034
	Sat	.108	.038	8.014	1	.005	1.114	1.034	1.201
	Temp	-.188	.303	.387	1	.534	.828	.458	1.499
	[Sex=זכר]	-.040	.331	.014	1	.905	.961	.502	1.841
	[Sex=נקבה]	0[b]	.	.	0	.	.	.	.
	[IHD=0]	.346	.622	.309	1	.579	1.413	.417	4.785
	[IHD=1]	0[b]	.	.	0	.	.	.	.
	[HTN=0]	.093	.459	.041	1	.839	1.098	.447	2.699
	[HTN=1]	0[b]	.	.	0	.	.	.	.
	[HLP=0]	-.652	.369	3.129	1	.077	.521	.253	1.073
	[HLP=1]	0[b]	.	.	0	.	.	.	.
	[PVD=0]	12.015	869.461	.000	1	.989	165221.229	.000	.[c]
	[PVD=1]	0[b]	.	.	0	.	.	.	.
	[HF=0]	-1.190	1.123	1.123	1	.289	.304	.034	2.748
	[HF=1]	0[b]	.	.	0	.	.	.	.
	[CVA=0]	.725	1.082	.449	1	.503	2.065	.248	17.223
	[CVA=1]	0[b]	.	.	0	.	.	.	.
	[AF=0]	-.681	.901	.571	1	.450	.506	.086	2.962
	[AF=1]	0[b]	.	.	0	.	.	.	.
	[other_medical_background=]	-17.730	4073.305	.000	1	.997	1.994E-8	.000	.[c]

	[other_medical_background=0]	-18.952	4073.304	.000	1	.996	5.877E-9	.000	.[c]
	[other_medical_background=AS]	-33.734	4841.108	.000	1	.994	2.237E-15	.000	.[c]
	[other_medical_background=BIOPROSTHETIC VALVE]	-18.568	5017.839	.000	1	.997	8.634E-9	.000	.[c]
	[other_medical_background=hocm]	-27.410	4841.108	.000	1	.995	1.247E-12	.000	.[c]
	[other_medical_background=HOCM]	-16.872	4073.305	.000	1	.997	4.704E-8	.000	.[c]
	[other_medical_background=MR]	.736	5760.523	.000	1	1.000	2.088	.000	.[c]
	[other_medical_background=ms]	17.118	6326.780	.000	1	.998	27176063.088	.000	.[c]
	[other_medical_background=pacemaker]	15.332	6147.173	.000	1	.998	4554454.550	.000	.[c]
	[other_medical_background=PACEMAKER]	4.843	5760.523	.000	1	.999	126.798	.000	.[c]
	[other_medical_background=pericardial effusion]	-30.668	4841.108	.000	1	.995	4.797E-14	.000	.[c]
	[other_medical_background=PERICARDIAL EFFUSION]	-31.902	4841.108	.000	1	.995	1.397E-14	.000	.[c]
	[other_medical_background=PHTN]	-32.174	4462.575	.000	1	.994	1.065E-14	.000	.[c]
	[other_medical_background=PROSTHETIC VALVE]	-36.451	4841.108	.000	1	.994	1.478E-16	.000	.[c]
	[other_medical_background=PULMONARY HTN]	-34.115	4450.551	.000	1	.994	1.527E-15	.000	.[c]
	[other_medical_background=RHD]	-31.564	4841.108	.000	1	.995	1.958E-14	.000	.[c]
	[other_medical_background=SSS]	14.180	6326.781	.000	1	.998	1439260.161	.000	.[c]
	[other_medical_background=SVT]	-15.03	4073.305	.000	1	.997	2.950E-7	.000	.[c]

		6						
	[other_medical_background=TOF; PHTN]	0[b]	.	.	0	.	.	.	.
	[CKD=0]	12.607	1319.994	.000	1	.992	298654.929	.000	.[c]
	[CKD=1]	10.727	1319.995	.000	1	.994	45580.331	.000	.[c]
	[CKD=2]	0[b]	.	.	0	.	.	.	.
	[LIVER DISEASE=0]	0[b]	.	.	0	.	.	.	.
	[DIABETES=0]	-.507	.406	1.563	1	.211	.602	.272	1.333
	[DIABETES=1]	0[b]	.	.	0	.	.	.	.
	[VTE=0]	1.583	.836	3.583	1	.058	4.871	.945	25.099
	[VTE=1]	0[b]	.	.	0	.	.	.	.
	[LUNG_DISEASE=0]	11.005	3950.929	.000	1	.998	60155.645	.000	.[c]
	[LUNG_DISEASE=1]	8.775	3950.929	.000	1	.998	6468.403	.000	.[c]
	[LUNG_DISEASE=2]	11.612	3950.929	.000	1	.998	110437.157	.000	.[c]
	[LUNG_DISEASE=3]	31.158	5674.650	.000	1	.996	34033003178518.266	.000	.[c]
	[LUNG_DISEASE=4]	10.777	5412.843	.000	1	.998	47885.980	.000	.[c]
	[LUNG_DISEASE=5]	0[b]	.	.	0	.	.	.	.
	[HYPOTHYROIDISM=.00]	-15.637	2616.204	.000	1	.995	1.618E-7	.000	.[c]
	[HYPOTHYROIDISM=1.00]	0[b]	.	.	0	.	.	.	.
	[other_MEDICAL_BACKGROUND=]	12.579	2616.204	.000	1	.996	290344.319	.000	.[c]
	[other_MEDICAL_BACKGROUND=0]	13.538	2616.204	.000	1	.996	757807.427	.000	.[c]
	[other_MEDICAL_BACKGROUND=ADRENAL INSUFFICEIANCY]	.959	3699.870	.000	1	1.000	2.608	.000	.[c]
	[other_MEDICAL_BACKGROUND=ALZHEIMER]	31.091	4841.109	.000	1	.995	31806303151087.780	.000	.[c]
	[other_MEDICAL	-2.305	3699.8	.000	1	1.000	.100	.000	.[c]

	_BACKGROUND =anemia]		70						
	[other_MEDICAL _BACKGROUND =ANEMIA]	-.237	2982.0 23	.000	1	1.000	.789	.000	.[c]
	[other_MEDICAL _BACKGROUND =AORTIC THROMBUS]	-1.323	3699.8 70	.000	1	1.000	.266	.000	.[c]
	[other_MEDICAL _BACKGROUND =APLA]	-.952	3699.8 70	.000	1	1.000	.386	.000	.[c]
	[other_MEDICAL _BACKGROUND =APLA, ADDISON]	.767	3699.8 70	.000	1	1.000	2.152	.000	.[c]
	[other_MEDICAL _BACKGROUND =ARTHRITIS]	-3.651	3699.8 70	.000	1	.999	.026	.000	.[c]
	[other_MEDICAL _BACKGROUND =BIPOLAR]	34.36 3	4841.1 09	.000	1	.994	83920645 5016787. 600	.000	.[c]
	[other_MEDICAL _BACKGROUND =CUSHING]	3.147	3699.8 71	.000	1	.999	23.256	.000	.[c]
	[other_MEDICAL _BACKGROUND =CVA; FIBROMUSCULA R DYSPLASIA]	31.76 7	4841.1 09	.000	1	.995	62576889 721891.0 70	.000	.[c]
	[other_MEDICAL _BACKGROUND =DEMENTIA]	0[b]	.	.	0	.	.	.	.
	[other_MEDICAL _BACKGROUND =DVT]	.579	3006.1 25	.000	1	1.000	1.784	.000	.[c]
	[other_MEDICAL _BACKGROUND =DVT, PE]	.611	3699.8 70	.000	1	1.000	1.843	.000	.[c]
	[other_MEDICAL _BACKGROUND =epilepsy]	- 19.88 5	5133.6 34	.000	1	.997	2.311E-9	.000	.[c]
	[other_MEDICAL _BACKGROUND =EPILEPSY]	1.850	3105.0 79	.000	1	1.000	6.361	.000	.[c]
	[other_MEDICAL _BACKGROUND =factor II mutation]	1.479	3699.8 70	.000	1	1.000	4.389	.000	.[c]

	[other_MEDICAL_BACKGROUND=Factor V L]	-17.962	3699.870	.000	1	.996	1.583E-8	.000	.[c]
	[other_MEDICAL_BACKGROUND=factor V leiden]	-.633	3699.870	.000	1	1.000	.531	.000	.[c]
	[other_MEDICAL_BACKGROUND=FALLS]	31.128	4841.109	.000	1	.995	33005096444429.094	.000	.[c]
	[other_MEDICAL_BACKGROUND=FMF]	1.407	3699.870	.000	1	1.000	4.083	.000	.[c]
	[other_MEDICAL_BACKGROUND=GVHD]	17.460	4531.398	.000	1	.997	38259853.232	.000	.[c]
	[other_MEDICAL_BACKGROUND=HYPOTHYRODISM]	-3.188	5906.350	.000	1	1.000	.041	.000	.[c]
	[other_MEDICAL_BACKGROUND=hypothyroidism]	-19.246	4277.048	.000	1	.996	4.380E-9	.000	.[c]
	[other_MEDICAL_BACKGROUND=HYpothyroidism]	1.298	3699.871	.000	1	1.000	3.662	.000	.[c]
	[other_MEDICAL_BACKGROUND=HYPOTHYROIDISM]	-2.293	3699.871	.000	1	1.000	.101	.000	.[c]
	[other_MEDICAL_BACKGROUND=hypothyroidism; anemia]	2.738	5232.407	.000	1	1.000	15.460	.000	.[c]
	[other_MEDICAL_BACKGROUND=HYPOTHYROIDOSM]	.973	5232.408	.000	1	1.000	2.647	.000	.[c]
	[other_MEDICAL_BACKGROUND=IBD]	.027	2904.755	.000	1	1.000	1.027	.000	.[c]
	[other_MEDICAL_BACKGROUND=IVDU]	14.819	2616.204	.000	1	.995	2728543.587	.000	.[c]
	[other_MEDICAL_BACKGROUND=KIDNEY TRANSPLANT]	21.011	4531.398	.000	1	.996	1334058481.327	.000	.[c]
	[other_MEDICAL_BACKGROUND	.890	3699.870	.000	1	1.000	2.436	.000	.[c]

	=MASSIVE PE]								
	[other_MEDICAL _BACKGROUND =MENINGIOM; HYPOTHYROIDISM]	-16.234	4531.397	.000	1	.997	8.906E-8	.000	.[c]
	[other_MEDICAL _BACKGROUND =MENINGIOMA]	.992	3699.870	.000	1	1.000	2.696	.000	.[c]
	[other_MEDICAL _BACKGROUND =MENINGITIS]	17.011	3893.572	.000	1	.997	24427281.464	.000	.[c]
	[other_MEDICAL _BACKGROUND =MGUS; hypothyroidism]	-13.179	4531.398	.000	1	.998	1.891E-6	.000	.[c]
	[other_MEDICAL _BACKGROUND =MICROVILLOUS ATROPHY]	.195	3699.870	.000	1	1.000	1.215	.000	.[c]
	[other_MEDICAL _BACKGROUND =migrain]	.183	3699.870	.000	1	1.000	1.201	.000	.[c]
	[other_MEDICAL _BACKGROUND =MS]	18.742	4531.398	.000	1	.997	137839593.985	.000	.[c]
	[other_MEDICAL _BACKGROUND =obesity]	14.369	3800.659	.000	1	.997	1738847.139	.000	.[c]
	[other_MEDICAL _BACKGROUND =OBESITY]	-1.515	3000.355	.000	1	1.000	.220	.000	.[c]
	[other_MEDICAL _BACKGROUND =OBESITY; HYPOTHYROIDISM]	-17.781	4531.397	.000	1	.997	1.895E-8	.000	.[c]
	[other_MEDICAL _BACKGROUND =obesity; S/P gastric bypass]	-1.779	3699.870	.000	1	1.000	.169	.000	.[c]
	[other_MEDICAL _BACKGROUND =PAEKINSONS]	33.505	4841.109	.000	1	.994	355641764964160.900	.000	.[c]
	[other_MEDICAL _BACKGROUND =parkinson]	-.250	3125.247	.000	1	1.000	.778	.000	.[c]
	[other_MEDICAL _BACKGROUND	-3.285	3699.870	.000	1	.999	.037	.000	.[c]

	=PARKINSON]								
	[other_MEDICAL_BACKGROUND =PE]	31.510	4841.109	.000	1	.995	48357472293440.920	.000	.[c]
	[other_MEDICAL_BACKGROUND =PEMPHIGUS]	-.525	3699.870	.000	1	1.000	.592	.000	.[c]
	[other_MEDICAL_BACKGROUND =PET]	17.438	4531.398	.000	1	.997	37430495.511	.000	.[c]
	[other_MEDICAL_BACKGROUND =PMR]	50.551	5502.805	.000	1	.993	8993600581201946000000.000	.000	.[c]
	[other_MEDICAL_BACKGROUND =POLycythemia vera;]	47.696	5502.805	.000	1	.993	5179514217409371000000.000	.000	.[c]
	[other_MEDICAL_BACKGROUND =polycythemia vera; hypothyrodism]	1.272	5232.408	.000	1	1.000	3.568	.000	.[c]
	[other_MEDICAL_BACKGROUND =PROTEIN c DEF]	-18.823	3699.870	.000	1	.996	6.688E-9	.000	.[c]
	[other_MEDICAL_BACKGROUND =PSHYCIATRIC]	-.036	3699.870	.000	1	1.000	.965	.000	.[c]
	[other_MEDICAL_BACKGROUND =PSORIASIS]	1.460	3699.870	.000	1	1.000	4.307	.000	.[c]
	[other_MEDICAL_BACKGROUND =PUD]	30.934	4841.109	.000	1	.995	27199490926415.086	.000	.[c]
	[other_MEDICAL_BACKGROUND =RA]	-.592	2960.630	.000	1	1.000	.553	.000	.[c]
	[other_MEDICAL_BACKGROUND =REHUMATOID ARTHRITIS]	2.908	3699.870	.000	1	.999	18.321	.000	.[c]
	[other_MEDICAL_BACKGROUND =SARCOIDOSIS]	-.827	3699.870	.000	1	1.000	.437	.000	.[c]
	[other_MEDICAL_BACKGROUND =SCHIZOPHREN	-.680	3103.236	.000	1	1.000	.507	.000	.[c]

	IA]								
	[other_MEDICAL _BACKGROUND =schizoprenia; epilepsy]	.347	3699.870	.000	1	1.000	1.414	.000	.[c]
	[other_MEDICAL _BACKGROUND =SCLERODERMA]	15.523	4111.524	.000	1	.997	5514957.782	.000	.[c]
	[other_MEDICAL _BACKGROUND =sjogren]	1.605	3699.870	.000	1	1.000	4.979	.000	.[c]
	[other_MEDICAL _BACKGROUND =TARDIVE DYSKINESIA]	15.651	4531.398	.000	1	.997	6265445.623	.000	.[c]
	[other_MEDICAL _BACKGROUND =thyrotoxicosis]	-1.585	3699.870	.000	1	1.000	.205	.000	.[c]
	[other_MEDICAL _BACKGROUND =ULCERATIVE COLITIS]	29.242	4841.110	.000	1	.995	5008475320815.528	.000	.[c]
	[other_MEDICAL _BACKGROUND =WEGENER]	0[b]	.	.	0	.	.	.	.
	[BB=0]	1.282	.479	7.177	1	.007	3.605	1.411	9.210
	[BB=1]	0[b]	.	.	0	.	.	.	.
	[ACEI=0]	.462	.536	.740	1	.390	1.586	.554	4.540
	[ACEI=1]	0[b]	.	.	0	.	.	.	.
	[ARB=0]	.347	.564	.378	1	.538	1.415	.468	4.272
	[ARB=1]	0[b]	.	.	0	.	.	.	.
	[diuretic=0]	-.050	.636	.006	1	.938	.952	.274	3.310
	[diuretic=1]	0[b]	.	.	0	.	.	.	.
	[MRA=0]	1.607	2.132	.568	1	.451	4.988	.076	325.873
	[MRA=1]	0[b]	.	.	0	.	.	.	.
	[alpha agonist=0]	12.057	1267.032	.000	1	.992	172290.027	.000	.[c]
	[alpha agonist=1]	0[b]	.	.	0	.	.	.	.
	[alpha bloker=0]	-1.042	.692	2.266	1	.132	.353	.091	1.370
	[alpha bloker=1]	0[b]	.	.	0	.	.	.	.
	[CCB=0]	-.058	.510	.013	1	.910	.944	.348	2.563
	[CCB=1]	0[b]	.	.	0	.	.	.	.
	[OTHER_MEDICATIONS=]	17.604	2616.204	.000	1	.995	44182225.000	.000	.[c]
	[OTHER_MEDICATIONS=0]	17.673	2616.204	.000	1	.995	47329369.922	.000	.[c]
	[OTHER_MEDIC	36.63	4841.1	.000	1	.994	81343715	.000	.[c]

	ATIONS=1]	5	10				89111011.000		
	[OTHER_MEDICATIONS=ANTIPSYCHOTICS]	17.650	4058.890	.000	1	.997	46249368.406	.000	.[c]
	[OTHER_MEDICATIONS=BNZ]	18.800	4075.744	.000	1	.996	146128361.465	.000	.[c]
	[OTHER_MEDICATIONS=DEXA; KEPPRA; PHENYTOIN]	36.040	4841.109	.000	1	.994	4486872096080437.500	.000	.[c]
	[OTHER_MEDICATIONS=dexamethasone]	3.512	3194.659	.000	1	.999	33.520	.000	.[c]
	[OTHER_MEDICATIONS=DEXAMETHASONE]	2.938	2883.641	.000	1	.999	18.876	.000	.[c]
	[OTHER_MEDICATIONS=DEXAMETHASONE; KEYTRUDA]	6.649	3699.870	.000	1	.999	772.256	.000	.[c]
	[OTHER_MEDICATIONS=DIGOXIN]	.522	6680.738	.000	1	1.000	1.686	.000	.[c]
	[OTHER_MEDICATIONS=eltroxin]	35.959	3383.582	.000	1	.992	4136732912736249.000	.000	.[c]
	[OTHER_MEDICATIONS=ELTROXIN]	5.835	3699.870	.000	1	.999	341.977	.000	.[c]
	[OTHER_MEDICATIONS=ENTECAVIR]	4.173	3699.870	.000	1	.999	64.939	.000	.[c]
	[OTHER_MEDICATIONS=EUTHYROX]	0[b]	.	.	0	.	.	.	.
	[OTHER_MEDICATIONS=Halidol, Assival]	35.438	4841.109	.000	1	.994	2458035396014045.000	.000	.[c]
	[OTHER_MEDICATIONS=hydralazine]	0[b]	.	.	0	.	.	.	.
	[OTHER_MEDICATIONS=hydroxyurea]	0[b]	.	.	0	.	.	.	.
	[OTHER_MEDICATIONS=IMURAN, PREDNISON]	0[b]	.	.	0	.	.	.	.
	[OTHER_MEDIC	16.10	3966.9	.000	1	.997	9876223.	.000	.[c]

	ATIONS=IRON]	6	86				855		
	[OTHER_MEDICATIONS=keppra, tegretol, vimpat, luminal]	39.119	5761.833	.000	1	.995	975765158134255 04.000	.000	.[c]
	[OTHER_MEDICATIONS=KEPRRA]	4.998	3699.871	.000	1	.999	148.097	.000	.[c]
	[OTHER_MEDICATIONS=LABA; PREDNISON]	-14.944	5502.805	.000	1	.998	3.234E-7	.000	.[c]
	[OTHER_MEDICATIONS=LAMA]	8.764	3699.871	.000	1	.998	6396.730	.000	.[c]
	[OTHER_MEDICATIONS=LAMA, ICS]	9.480	3699.871	.000	1	.998	13093.190	.000	.[c]
	[OTHER_MEDICATIONS=LAMA; METHADONE]	36.441	4841.110	.000	1	.994	6700303722583692.000	.000	.[c]
	[OTHER_MEDICATIONS=LTA, ICS]	4.218	3699.870	.000	1	.999	67.869	.000	.[c]
	[OTHER_MEDICATIONS=NITRATES]	4.467	3699.870	.000	1	.999	87.071	.000	.[c]
	[OTHER_MEDICATIONS=Oral contraceptive]	2.795	3699.870	.000	1	.999	16.364	.000	.[c]
	[OTHER_MEDICATIONS=phenytoin]	36.753	4841.109	.000	1	.994	9156325744191112.000	.000	.[c]
	[OTHER_MEDICATIONS=prednison]	16.111	3950.930	.000	1	.997	9927948.136	.000	.[c]
	[OTHER_MEDICATIONS=PREDNISON]	18.024	2616.204	.000	1	.995	67224985.396	.000	.[c]
	[OTHER_MEDICATIONS=PREDNISON, CYCLOSPORIN]	0[b]	.	.	0	.	.	.	.
	[OTHER_MEDICATIONS=PREDNISON; MTX]	4.523	3081.572	.000	1	.999	92.067	.000	.[c]
	[OTHER_MEDICATIONS=PREDNISON; TACROLIMUS; MYFORTIC]	2.020	3699.870	.000	1	1.000	7.537	.000	.[c]

	[OTHER_MEDICATIONS=PREDNISON;TACROLIMUS]	0[b]	.	.	0	.	.	.	.
	[OTHER_MEDICATIONS=prednisone]	6.049	3699.870	.000	1	.999	423.596	.000	.[c]
	[OTHER_MEDICATIONS=SILDENAFIL]	5.525	3699.871	.000	1	.999	250.925	.000	.[c]
	[OTHER_MEDICATIONS=SIMOVIL]	34.325	3867.276	.000	1	.993	807677519264686.800	.000	.[c]
	[OTHER_MEDICATIONS=SNRI]	0[b]	.	.	0	.	.	.	.
	[OTHER_MEDICATIONS=STEROIDS]	4.030	3699.871	.000	1	.999	56.282	.000	.[c]
	[OTHER_MEDICATIONS=TAMSULOSIN, CLONEX]	0[b]	.	.	0	.	.	.	.
	[OTHER_MEDICATIONS=TEGRETOL]	68.343	6553.321	.000	1	.992	4798608340925472000000000000000.000	.000	.[c]
	[OTHER_MEDICATIONS=tegretol; BNZ]	0[b]	.	.	0	.	.	.	.
	[OTHER_MEDICATIONS=thyroxin]	18.559	2616.204	.000	1	.994	114847978.595	.000	.[c]
	[OTHER_MEDICATIONS=THYROXIN]	18.106	2616.204	.000	1	.994	72968600.519	.000	.[c]
	[OTHER_MEDICATIONS=thyroxin; hydroxyurea]	0[b]	.	.	0	.	.	.	.
	[OTHER_MEDICATIONS=TRAMADOL]	3.373	3699.870	.000	1	.999	29.167	.000	.[c]
	[OTHER_MEDICATIONS=verapamil]	0[b]	.	.	0	.	.	.	.
	[ASPIRIN=0]	.665	.490	1.844	1	.175	1.945	.745	5.079
	[ASPIRIN=1]	0[b]	.	.	0	.	.	.	.
	[PAVIX=0]	-.876	1.063	.678	1	.410	.417	.052	3.348
	[PAVIX=1]	0[b]	.	.	0	.	.	.	.
	[DAPT=.00]	-2.486	1.754	2.008	1	.156	.083	.003	2.591

	[DAPT=1.00]	0[b]	.	.	0	.	.	.	.
	[AC=0]	-1.246	1.396	.797	1	.372	.288	.019	4.436
	[AC=1]	-1.176	1.478	.632	1	.426	.309	.017	5.595
	[AC=2]	3.962	2.393	2.740	1	.098	52.537	.482	5724.903
	[AC=3]	0[b]	.	.	0	.	.	.	.
	[CLEXANE=0]	0[b]	.	.	0	.	.	.	.
	[CLEXANE=1]	0[b]	.	.	0	.	.	.	.
	[DOACS=0]	0[b]	.	.	0	.	.	.	.
	[DOACS=1]	0[b]	.	.	0	.	.	.	.
	[VKA=0]	0[b]	.	.	0	.	.	.	.
	[VKA=1]	0[b]	.	.	0	.	.	.	.
	[provokation other than malignancy=0]	-18.444	.427	1862.532	1	.000	9.767E-9	4.227E-9	2.257E-8
	[provokation other than malignancy=1]	-17.521	.000	.	1	.	2.459E-8	2.459E-8	2.459E-8
	[provokation other than malignancy=2]	0[b]	.	.	0	.	.	.	.
	[is there a background Dx of malig=0]	-.899	.377	5.694	1	.017	.407	.194	.852
	[is there a background Dx of malig=1]	0[b]	.	.	0	.	.	.	.

a. The reference category is: .00.

b. This parameter is set to zero because it is redundant.

c. Floating point overflow occurred while computing this statistic. Its value is therefore set to system missing.
